# A Prediction Model for Acute Kidney Injury in Adult Patients With Minimal Change Disease

**DOI:** 10.3389/fmed.2022.862160

**Published:** 2022-05-24

**Authors:** Chen Yang, Chen Yang, Shu-Peng Lin, Pu Chen, Jie Wu, Jin-Ling Meng, Shuang Liang, Feng-Ge Zhu, Yong Wang, Zhe Feng, Xiang-Mei Chen, Guang-Yan Cai

**Affiliations:** ^1^School of Medicine, Nankai University, Tianjin, China; ^2^Department of Nephrology, First Medical Center of Chinese PLA General Hospital, Nephrology Institute of the Chinese PLA, State Key Laboratory of Kidney Diseases, National Clinical Research Center for Kidney Diseases, Beijing Key Laboratory of Kidney Disease Research, Beijing, China; ^3^Department of Nephrology, Cangzhou Center Hospital, Cangzhou, China

**Keywords:** nomogram, acute kidney injury (AKI), minimal change disease, prediction model, nephrotic syndrome

## Abstract

**Background:**

Early prediction of acute kidney injury (AKI) can allow for timely interventions, but there are still few methods that are easy and convenient to apply in predicting AKI, specially targeted at patients with minimal change disease (MCD). Motivated by this, we aimed to develop a predicting model for AKI in patients with MCD within the KDIGO criteria.

**Methods:**

Data on 401 hospitalized adult patients, whose biopsy was diagnosed as MCD from 12/31/2010 to 15/7/2021, were retrospectively collected. Among these data, patients underwent biopsy earlier formed the training set (*n* = 283), while the remaining patients formed the validation set (*n* = 118). Independent risk factors associated with AKI were analyzed. From this, the prediction model was developed and nomogram was plotted.

**Results:**

AKI was found in 55 of 283 patients (19%) and 15 of 118 patients (13%) in the training and validation cohorts, respectively. According to the results from lasso regression and logistic regression, it was found that four factors, including mean arterial pressure, serum albumin, uric acid, and lymphocyte counts, were independent of the onset of AKI. Incorporating these factors, the nomogram achieved a reasonably good concordance index of 0.84 (95%CI 0.77–0.90) and 0.75 (95%CI 0.62–0.87) in predicting AKI in the training and validation cohorts, respectively. Decision curve analysis suggested clinical benefit of the prediction models.

**Conclusions:**

Our predictive nomogram provides a feasible approach to identify high risk MCD patients who might develop AKI, which might facilitate the timely treatment.

## Introduction

Minimal change disease (MCD) is one of the main causes of nephrotic syndrome (NS). Microscopically, it reveals a normal appearance of glomeruli by light microscopy, and also reveals negative immunofluorescence and foot-process fusion by electron microscopy (1, 2). Though people of all ages may develop MCD, previous studies suggest that younger children are more susceptible (2). Particularly, for patients with NS, it accounts for 70–90% in younger children, 50% in older children and 10–20% in adults (3–5).

Since MCD usually proceeds in a moderate manner, few patients would reach end-stage renal disease (ESRD) (6–8), but approximately one-fifth to one-third of adult MCD patients may develop AKI instead (1). Hence, AKI has been perceived as a common complication among adult MCD patients. Due to the lack of effective pharmacotherapeutic, it often imposes severe public health burden on patients. Therefore, from the perspective of patients, prevention of AKI is more desirable than the costly treatment. Motivated by this, we shall develop an effective prediction model to make earlier identification.

In the literature, many research efforts have sought to predict the occurrence of AKI. Several prediction models have been developed, focusing on the post-operative, the severe sepsis and many other kinds of patients (9–13). However, what is missing is a model for prediction AKI among patients with MCD. For MCD patients who develop concurrent AKI, their clinical characteristics including sex, age, proteinuria, serum albumin and blood pressure, are comparable (1). However, a simple but practical method for predicting AKI, especially for patients with MCD, is still lacking.

Considering that inpatient AKI usually occurs outside the hospital, nephrologists are in need of a simple tool to detect high-risk patients. Therefore, we performed a retrospective study of patients with biopsy-proven MCD from 2011 to 2021. For the sake of clinical practicability and convenience, we focus mainly on static variables with some routine laboratory inspection, and establish a simple but targeted model to predict the occurrence of AKI. Particularly, we propose a prediction model based on the training set including 283 patients, and verified its performance using the validation cohorts of 118 patients.

## Materials and Methods

### Patients

This is a retrospective study of adults aged 18 and older with renal biopsy diagnosed MCD. Patients with at least two creatinine values, admitted from 12/31/2010 to 15/7/2021, were considered, while those lacking the two values were excluded. Patients with previous serum creatinine level >110 μmol/L (upper normal limit), or estimated glomerular filtration rate (eGFR, EPI Equation) <60 ml/min/1.73 m^2^ before the onset of AKI were also excluded. We used the data of patients enrolled from 12/31/2010 to 12/31/2017 for training the model and those enrolled from 1/1/2018 to 7/15/2021 for validation. Inclusion and exclusion criteria are the same as for the training cohort. Ethics approval was obtained from the Research Ethics Committee of Chinese PLA General Hospital.

### Variable Ascertainment

Demographics, vital signs, laboratory data and treatment were collected from patients' medical records for analysis. In particular, we extracted the following indicators: age, sex, blood pressure, laboratory indices, including 24-h urine protein, serum albumin, serum creatinine, uric acid, blood glucose, C3, C4, eGFR, total cholesterol, triglyceride, IgA, IgE, IgG, IgM, hemoglobin, white blood cells, neutrophil counts, lymphocyte counts, CRP, treatment, including glucocorticoids, immunosuppressive agents, renin angiotensin system inhibitors (RASI) and AKI stage. Laboratory data was collected from the time points before the AKI occurrence within 1 week. If there were multiple measurements, we took the first value of the variable.

### Clinical Definitions

Acute kidney injury was defined as per the Kidney Disease: Improving Global Outcomes (KDIGO) creatinine criteria (14). Due to few urine output data, we did not select urine output as the evaluation criteria. AKI was therefore identified by the 1.5 times increase in serum creatinine over the 7-day period compared to the lowest, and serum creatinine increased by 0.3 mg/dl within 48 h. The creatinine value at baseline was derived from either outpatient or inpatient laboratory data.

### Statistical Methods

We applied descriptive analysis to characterize enrolled MCD patients who have developed AKI. Missing data was limited to laboratory values and represented <5% of all observations. Where the data was missing, the value was imputed using the mean value for a patient of that group by AKI status. Data was presented as mean ± standard deviation (SD) for measurement data and percent-age for enumeration count data. The statistical significance level was set at *P* < 0.05. Data were compared using student's *t*-test, Wilcoxon's test or chi-squared tests as appropriate. The least absolute shrinkage and selection operator (LASSO) logistic regression model was used with penalty parameter tuning that was conducted by 10-fold cross-validation based on minimum criteria. Predictive features selected by LASSO were input into the binary logistic regression. The nomogram was performed by a simple selection process using a threshold of *P* < 0.05. The model's prediction ability was measured by the area under the receiver operating characteristic curve (AUROC) (15). A decision curve analysis was performed, aiming to determine the clinical net benefit of probability thresholds for a possible clinical consequence and reliability of the model according to the method of Vickers et al. (16, 17). All statistical tests were established with R software (version 3.6.3; http://www.R-project.org).

## Results

### Clinicopathologic Characteristics

**Figure 1:**
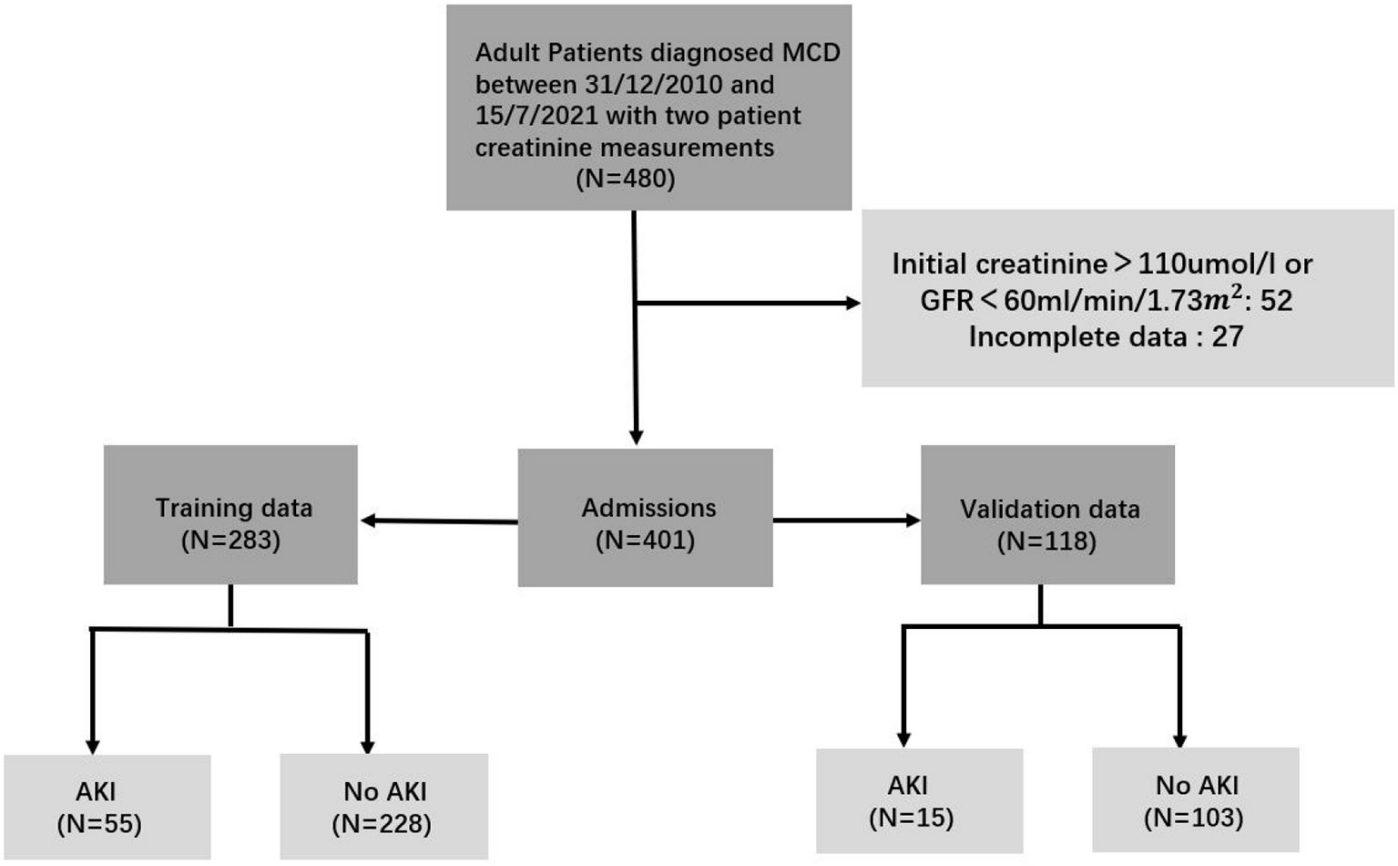
Flow diagram of the patient cohort. To build up the prediction model, the study population was divided into training and validation cohorts.

We identified 401 qualified patients for the analyses, of which 283 patients from 12/31/2010 to 12/31/2017 formed the training cohort, while the remaining 118 from 1/1/2018 to 15/7/2021 constituted the validation cohort (see Figure 1). More detailed characteristics of patients were displayed in Table 1. They were similar in the laboratory parameters. However, patients were more likely to be female, the incidence of AKI and glucocorticoids application was higher in the training group. And tacrolimus applicated more in the validation group. Besides, there was no significant concomitant conditions between the two cohort. There were 55 patients (19%) and 15 patients (13%) occurred AKI in the training and validation group, respectively. Stage1 AKI were more prevalent in those patients. The median time from admission to diagnosis AKI was 2 days in AKI patients.

**Table 1:** Baseline characteristics of patient groups.

**Variable**	**Cohort**		***P*-Value**
	**Training** **(*N* = 283)**	**Validation** **(*N* = 118)**	
**Demographics**			
Age, year	39.5 ± 14.2	37.6 ± 15.4	0.148
Sex, male (%)	139 (49.1)	78 (66.1%)	0.002
**Blood pressure**			
Mean arterial pressure, mmHg	92.0 ± 10.6	92.9 ± 12.0	0.337
**Laboratory**			
24 h urine protein, g/day	5.6 ± 3.5	5.2 ± 3.3	0.360
Serum albumin, g/L	21.5 ± 6.2	21.1 ± 5.8	0.518
Serum creatinine, μmol/L	73.9 ± 15.6	76.0 ± 16.1	0.223
Uric acid, μmol/L	330.5 ± 104.4	360.5 ± 99.8	0.002
Blood glucose, mmol/L	4.7 ± 0.9	5.0 ± 1.5	0.004
Serum C3, mg/dl	123.7 ± 25.4	126.5 ± 26.1	0.285
Serum C4, mg/dl	31.0 ± 9.6	30.7 ± 11.0	0.479
GFR, ml/min/1.73 m^2^	109.1 ± 23.3	113.2 ± 26.5	0.291
Total cholesterol, mmol/L	9.1 ± 2.7	8.6 ± 2.5	0.070
Triglyceride, mmol/L	2.4 ± 1.2	2.8 ± 1.6	0.026
IgA, mg/dl	241.7 ± 90.2	239.3 ± 83.2	0.977
IgE, IU/ml	795.6 ± 1,681.0	685.1 ± 1,300.8	0.898
IgG, mg/dl	515.8 ± 299.2	464.2 ± 242.0	0.167
IgM, mg/dl	148.8 ± 81.5	128.6 ± 63.3	0.066
**Blood routine**			
Hemoglobin, g/L	141.6 ± 19.3	143.2 ± 20.5	0.493
White blood cell, *10^9^/L	7.3 ± 2.7	7.2 ± 2.3	0.502
Neutrophil counts, *10^9^/L	4.7 ± 2.6	4.4 ± 2.1	0.797
Lymphocyte counts, *10^9^/L	2.0 ± 0.8	2.1 ± 0.7	0.025
C-reactive protein, mg/dl	0.3 ± 0.5	0.2 ± 0.4	<0.001
**Treatment**			
Glucocorticoids, (%)	245 (86.6)	65 (55.1)	<0.001
Cyclophosphamide, (%)	1 (0.4)	2 (1.7)	0.204
Cyclosporine, (%)	5 (1.8)	1 (0.8)	0.439
Mycophenolate Mofetil, (%)	1 (0.4)	1 (0.8)	0.497
Tacrolimus, (%)	16 (5.7)	48 (40.7)	<0.001
Tripterygium wilfordii, (%)	2 (0.7)	1 (0.8)	0.644
RASI, (%)	78 (27.6)	31 (26.3)	0.791
**Concomitant conditions**			
Diabetes, (%)	9 (3.2)	5 (4.2)	0.396
Hypertension, (%)	47 (16.6)	16 (13.6)	0.431
Cardiovascular disease, (%)	4 (1.4)	2 (1.7)	0.339
Cerebrovascular disease, (%)	2 (0.7)	3 (2.5)	0.065
Old fracture, (%)	1 (0.4)	1 (0.8)	0.502
Peptic ulcer, (%)	3 (1.1)	1 (0.8)	0.662
Venous thromboembolism, (%)	6 (2.1)	3 (2.5)	0.523
Infections, (%)	34 (12.0)	14 (11.9)	0.557
Tuberculosis, (%)	6 (2.1)	2 (1.7)	0.566
Hepatitis B virus carrier, (%)	4 (1.4)	3 (2.5)	0.339
Neuropsychiatric problems, (%)	1 (0.4)	0 (0)	0.706
**Renal pathology**			
C3 deposition	2 (0.9)	0 (0)	1
C4 deposition	0 (0)	0 (0)	1
**AKI (any stage)**	55 (19.4)	15 (12.7)	0.114
AKI stage 1	40 (14.1)	8 (6.8)	0.043
AKI stage 2	11 (3.9)	6 (5.1)	0.592
AKI stage 3	4 (1.4)	1 (0.8)	1
Time from admission to diagnosis	2 (1.5)	4 (1.9)	0.225

*Note that data are presented as mean ± standard deviation (SD), median (interquartile range), and number of cases (percentage) as appropriate*.

*RASI, renin angiotensin system inhibitors*.

### Risk Factors for AKI in Patients With MCD

We first conducted a univariable analysis for possible factors associated with AKI. Briefly, AKI is more likely to occur in men, the older, and people with higher mean arterial pressure. Concerning laboratory studies, serum albumin and IgG were inversely correlated with imminent AKI, whereas serum creatinine, serum uric acid, C4, triglyceride, 24 h urine protein and IgE were all at play in AKI development. With respect to blood routine, we found that patients with a higher risk of AKI featured with higher neutrophil counts and C-reactive protein (CRP), while the contrary holds for the lymphocyte counts. To sum up, patients with AKI were tended to be older, higher disease activity and worser laboratory factors.

**Figure 2:**
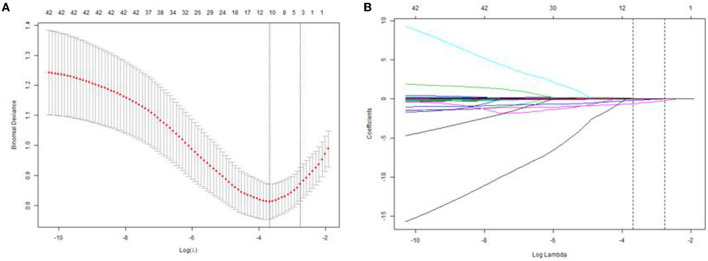
Texture feature selection using LASSO logistic regression and the predictive accuracy of the radiomics signature. **(A)** Selection of the tuning parameter (λ) in the LASSO model *via* 10-fold cross validation based on minimum criteria. The optimal λ value of 0.0577 with *log*(λ) = −2.85 was selected. **(B)** LASSO coefficient profiles of the 25 texture features. The dotted vertical line was plotted at the value selected using 10-fold cross-validation in **(A)**.

Subsequently, it was found that four factors, including mean arterial pressure, serum albumin, uric acid, and lymphocyte counts were selected from 25 clinical features (AKI stage included) based on the training cohort by LASSO regression model (Figures 2A,B). We further performed the binary logistic regression to confirm the independence. It was found that the four factors were still independently associated with risk of AKI, and their associated odds ratio in the training cohort were displayed in Table 2.

**Table 2:** Univariable and multivariate logistic regression analysis results.

**Variable**	**Univariable**			**Multivariate**		
	**β**	**OR (95% CI)**	***P*-Value**	**β**	**OR (95% CI)**	***P*-Value**
Mean arterial pressure, mmHg	0.05	1.051 (1.021–1.082)	0.001	0.05	1.046 (1.010–1.084)	0.011
Serum albumin, g/L	−0.12	0.883 (0.823–0.947)	<0.001	−0.13	0.878 (0.809–0.953)	0.002
Uric acid, μmol/L	0.01	1.009 (1.006–1.012)	<0.001	0.01	1.008 (1.004–1.011)	<0.001
Lymphocyte counts, *10^9^/L	−1.05	0.349 (0.206–0.592)	<0.001	−1.14	0.318 (0.173–0.587)	0.002

### Development and Validation of an AKI-Predicting Nomogram

**Figure 3:**
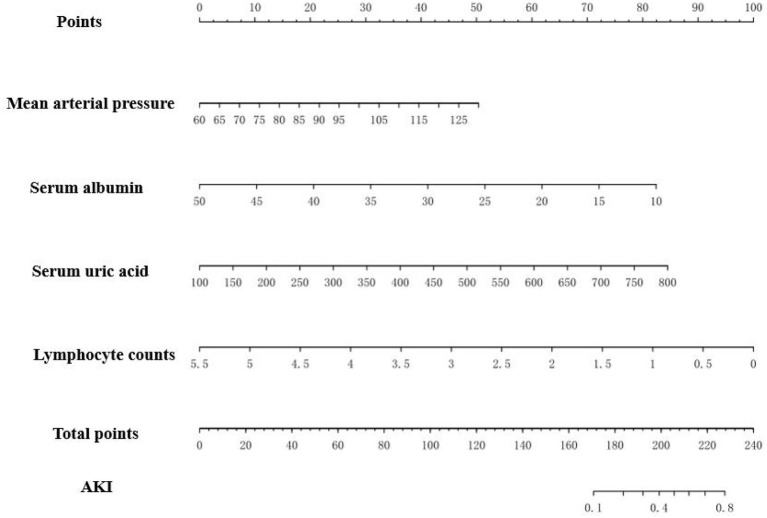
The nomogram to estimate the risk of AKI in MCD patients. To use the nomogram, search the position of each variable on the corresponding axis, draw a line to the points axis for the number of points, add the total points, and draw a line from the total points axis to determine the AKI probabilities at the lower line of the nomogram. For example, doctor checked an MCD patient blood pressure is 120/90 mmHg, and her laboratory test revealed serum albumin 20 g/L, serum urci acid 400 μmol/L, lymphocyte counts 2.5*10^9^/L. Based on the nomogram, her points were 29, 62, 36 and 55, respectively. The total points was 182 and the probability of AKI was 0.2.

The prediction model we proposed, which incorporates mean arterial pressure, serum albumin, uric acid and lymphocyte counts performed well in terms of imminent prediction of AKI. The scoring model was given by: −3.64259 +0.04533^*^mean arterial pressure −0.12975^*^ albumin +0.00760^*^ uric acid −1.14485^*^ lymphocyte counts. Based on it, the nomogram was plotted (Figure 3).

**Figure 4:**
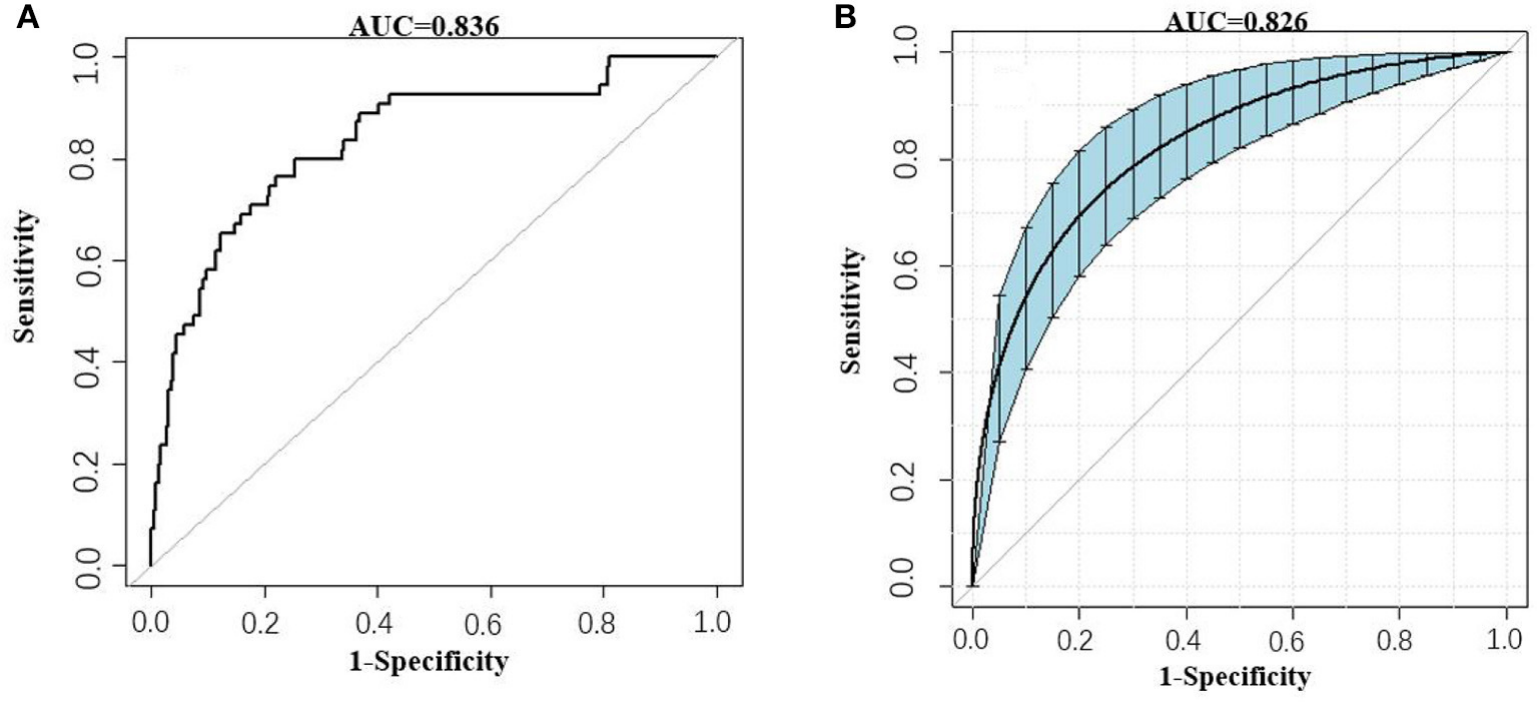
ROC curves of the AKI model. **(A)** Prediction of AKI (AUC = 0.84 95%CI 0.77–0.90). **(B)** Prediction of AKI by the bootstrap (500 resample) method. Blue shading shows the bootstrap estimated 95% CI with the AUC (AUC = 0.83 95%CI 0.75–0.89).

**Figure 5:**
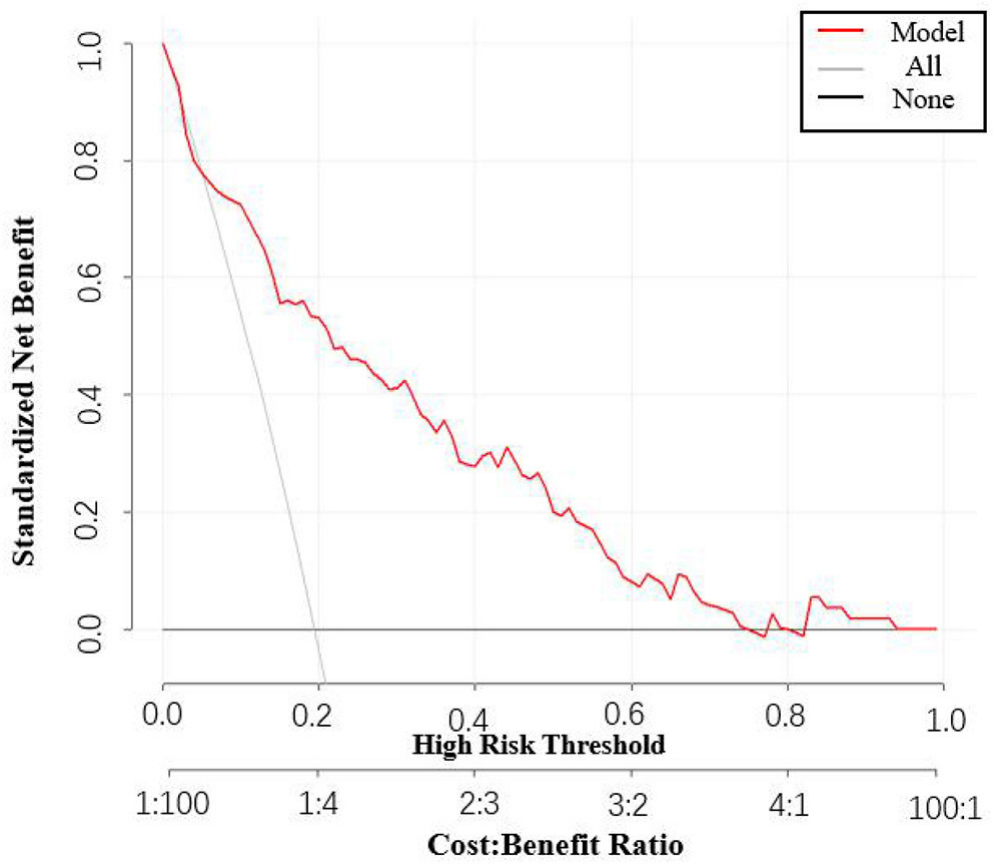
Decision curve analysis depicting the clinical efficiency in our cohort.

Besides, the performance of the nomogram was measured by ROC curves 0.84 (95%CI 0.77–0.90; Figure 4A). The nomogram's predictive accuracy was assessed by the bootstrap (500 resample) method, and only a slight change was observed for the ROC of the model (ROC = 0.83; Figure 4B) (18). Furthermore, we also examined the performance of the model with decision curves (Figure 5). The figure illustrates a well-calibrated model with a relatively high area under the nomogram curve. To further validate the efficacy of the model, we integrated the validation cohort that met the same inclusion criteria. The results showed that the ROC curves were 0.75 (95%CI 0.62–0.87; Table 3).

**Table 3:** Accuracy of the prediction score of the nomogram.

**Variable**	**Value**	
	**Training**	**Validation**
	**cohort**	**cohort**
Area under ROC curve, concordance index	0.84	0.75
Sensitivity, %	80.0	80.0
Specificity, %	74.6	67.0
Positive predictive value, %	43.1	26.1
Negative predictive value, %	93.9	95.8
Positive likelihood ratio	3.14	2.42
Negative likelihood ratio	0.27	0.30

*AKI, acute kidney injury; ROC, receiver operating characteristic*.

### Optimal Cutoff Value for Nomogram Score

There is a summary of sensitivity and specificity at different cutoff values (Table 4). At a cutoff value of ≥0.40, sensitivity is 47% and specificity is 93%. Although higher cutoff values result in higher specificity, sensitivity rapidly decreased to a point at which the model identifies only a third of patients in whom AKI may be omitted.

**Table 4:** Values of sensitivity, specificity, and predictive values of the nomogram scores at different cutoff values.

**Nomogram score**	**Sensitivity (%)**	**Specificity (%)**	**PPV (%)**	**NPV (%)**
≥0.2	76.4	77.6	45.2	93.2
≥0.3	65.5	86.4	53.7	91.2
≥0.4	47.3	93.0	61.9	88.0
≥0.5	36.4	96.1	69.0	86.2
≥0.6	27.3	96.9	68.2	84.7
≥0.7	12.7	99.1	77.8	82.5
≥0.8	7.3	99.6	80.0	81.7

*PPV, positive predictive value; NPV, negative predictive value*.

### Performance in Individual Patients

PPVs at different cutoff values at the AKI prevalence of 19%, which are associated with different sensitivities and specificities, are listed in Table 4. At a cutoff value of≥0.40, PPV is 62%. Although higher cutoff values might increase PPV marginally (to a maximum of 69%), it could cause the sensitivity to decrease.

## Discussion

Acute kidney injury is a common complication in adults with MCD, and the pathophysiological mechanism of AKI is still unclear yet. Although nephrologist have found several factors such as age, gender, albumin, urinary protein and people with a background of hypertension and renal microvascular lesions, are highly associated with AKI, there is still few effective and practical method for predicting AKI in MCD patients (1). In this study, we developed a model and proposed a nomogram to predict the onset of AKI, which is targeted on MCD patients. For practical convenience, we utilized only routine-tested variables to develop the simple model, and results showed that the model performed well in predicting AKI.

In our study, it is revealed that several variables have strong relationships with impending AKI. Particularly, when adopting LASSO regression and logistic analysis, four factors including mean arterial pressure, uric acid, albumin, and lymphocyte counts were significantly associated with the risk of AKI. Similar to previous studies, patients who developed AKI had higher mean arterial pressure, which is mainly due to renal ischemia (19). In laboratory factors, patients with AKI had significantly lower albumin levels than those without AKI. This may arise from fluid retention in the third interstitial space and insufficient effective circulating blood volume (20). In addition to the above factors, uric acid might be an effective predictive factor for AKI. Although uric acid has exhibited a strong relation with AKI model constructed for other diseases, it was rarely mentioned in MCD patients (21, 22). In our cohort, there was a significant difference between the AKI group and non-AKI group. High uric acid levels is deemed to be associated with an absence of intrarenal crystals, manifestation of tubular injury, macrophage infiltration and increased expression of inflammatory mediators, which could cause the onset of AKI (23).

There is another factor worthy of further discussion in our result, which is rarely considered in AKI prediction. In this study, we found that low level of lymphocyte counts was strong associated with the onset of AKI. Consistently, Tagawa et al. collected 445 AKI patients who underwent non-cardiac surgery and also found that lymphocyte counts were significantly lower in AKI groups (24). Similarly, Wang's et al. found that lymphocyte counts were independently associated with AKI after traumatic brain injury, and lymphocyte counts were incorporated into their final predictive nomogram (25). Concerning the results may correlate with infection, we also considered neutrophil counts and white blood cell counts to exclude the interaction between infection and renal function. The results showed that there was no significant difference between the two groups, implying that the decrease of lymphocytes counts may be less associated with infection. Nevertheless, previous study found that decrease in lymphocyte counts may due to aging or drop in kidney function, and prompt the shift to a memory profile and diverge in treg population (26). To sum up, both this paper and previous studies have proved that lymphocyte counts play an important role in predicting AKI.

In recent studies that predict the onset of AKI, predictive models and new biomarkers were frequently mentioned (27–30). In particular, electronic alerts have gained increasing popularity in predicting AKI due to its convenience and accuracy. The follow-up verification experiments, however, have seen inconsistent results using electronic alerts (31–33). In addition, the electronic alert system includes more variables than our model, such as previous medical history and other indicators, which leads to longer time for outpatient doctors to assess. More importantly, many new biomarkers have been proposed for detection, but some of these markers are much more troublesome to obtain in primary hospitals and increased medical expenses (34).

Since AKI is one of the common complications in MCD patients, early prediction and timely treatment could reduce the risk of it. Previous studies confirmed that AKI could be prevented by early interventions of individuals. Therefore, our primary goal is to use routine laboratory indicators to enable physicians to simply and quickly screen high-risk patients and assess the need for intervention. Among the existing various ways to predict AKI, the nomogram is a more intuitive and straightforward way, as it is more suitable for clinicians to present the model information in a simple and clear graphical manner. It also allows clinicians to quickly calculate the probability for a patient to develop AKI based on the clinical data of these patients.

Based on the above results, we further developed a predictive nomogram using the four risk factors mentioned above and validated its performance. It was found that the nomogram exhibited good predictive accuracy after internal validation (bootstrap). We also made a decision curve to assess the clinical benefit, and found that there was a relatively high area under the nomogram curve. Overall, it was demonstrated that the combination of these four indicators is a good predictor of AKI.

Despite the practical convenience of the proposed model, this study has several limitations. First, the data was collected from a single institution, and it was necessary to validate the results for other centers. Second, the study mainly included patients with serum creatinine lower than normal limit and GFR >60 ml/min/1.73 m^2^, which might decreased the generalizability of our findings. Third, the nomogram was performed based on retrospective cohort data, and prospective studies are needed to verify the accuracy further. Finally, despite the satisfactory performance of the proposed model, it is not far superior to other models that have been developed. We hope that our future studies could improve the predictive power of the model by combining biomarkers, enlarging the sample size and more.

In conclusion, this study presents a radiomics nomogram that incorporates four routine-test laboratory factors to estimate the risk of AKI in patients with MCD. As we known, it is the first predictive model, specially targeted at MCD patients. AND the model shows its potential of being conveniently used to facilitate the prediction of AKI.

## Data Availability Statement

The raw data supporting the conclusions of this article will be made available by the authors, without undue reservation.

## Ethics Statement

The studies involving human participants were reviewed and approved by Research Ethics Committee of Chinese PLA General Hospital. The patients/participants provided their written informed consent to participate in this study.

## Author Contributions

CY (1st author), ZF, and G-YC designed the study. ZF, G-YC, and X-MC revised and approved the analytical protocol. CY (2nd author) and S-PL collected the data. CY (1st author), PC, JW, J-LM, and F-GZ analyzed the data. YW and SL made the tables and figures. CY (1st author) first drafted the manuscript. S-PL, F-GZ, PC, JW, J-LM, SL, ZF, G-YC, and X-MC revised and amended the manuscript. All authors approved the final version of the manuscript.

## Funding

This study was supported by the Science and Technology Project of Beijing, China (D181100000118004), National Key Research and Development (R&D) Program of China (2018YFA0108803), and the Natural Science Foundation of China (NSFC) (82000675) for the support.

## Conflict of Interest

The authors declare that the research was conducted in the absence of any commercial or financial relationships that could be construed as a potential conflict of interest.

## Publisher's Note

All claims expressed in this article are solely those of the authors and do not necessarily represent those of their affiliated organizations, or those of the publisher, the editors and the reviewers. Any product that may be evaluated in this article, or claim that may be made by its manufacturer, is not guaranteed or endorsed by the publisher.
